# *In Vitro* Culture of the Insect Endosymbiont *Spiroplasma poulsonii* Highlights Bacterial Genes Involved in Host-Symbiont Interaction

**DOI:** 10.1128/mBio.00024-18

**Published:** 2018-03-20

**Authors:** Florent Masson, Sandra Calderon Copete, Fanny Schüpfer, Gonzalo Garcia-Arraez, Bruno Lemaitre

**Affiliations:** a Global Health Institute, School of Life Sciences, École Polytechnique Fédérale de Lausanne (EPFL), Lausanne, Switzerland; b Center for Integrative Genomics, Lausanne Genomic Technologies Facility, Lausanne, Switzerland; University of Hawaii at Manoa

**Keywords:** *Spiroplasma*, endosymbiosis, host-symbiont interaction

## Abstract

Endosymbiotic bacteria associated with eukaryotic hosts are omnipresent in nature, particularly in insects. Studying the bacterial side of host-symbiont interactions is, however, often limited by the unculturability and genetic intractability of the symbionts. *Spiroplasma poulsonii* is a maternally transmitted bacterial endosymbiont that is naturally associated with several *Drosophila* species. *S. poulsonii* strongly affects its host’s physiology, for example by causing male killing or by protecting it against various parasites. Despite intense work on this model since the 1950s, attempts to cultivate endosymbiotic *Spiroplasma in vitro* have failed so far. Here, we developed a method to sustain the *in vitro* culture of *S. poulsonii* by optimizing a commercially accessible medium. We also provide a complete genome assembly, including the first sequence of a natural plasmid of an endosymbiotic *Spiroplasma* species. Last, by comparing the transcriptome of the *in vitro* culture to the transcriptome of bacteria extracted from the host, we identified genes putatively involved in host-symbiont interactions. This work provides new opportunities to study the physiology of endosymbiotic *Spiroplasma* and paves the way to dissect insect-endosymbiont interactions with two genetically tractable partners.

## IMPORTANCE

The discovery of insect bacterial endosymbionts (maternally transmitted bacteria) has revolutionized the study of insects, suggesting novel strategies for their control. Most endosymbionts are strongly dependent on their host to survive, making them uncultivable in artificial systems and genetically intractable. *Spiroplasma poulsonii* is an endosymbiont of *Drosophila* that affects host metabolism, reproduction, and defense against parasites. By providing the first reliable culture medium that allows a long-lasting *in vitro* culture of *Spiroplasma* and by elucidating its complete genome, this work lays the foundation for the development of genetic engineering tools to dissect endosymbiosis with two partners amenable to molecular study. Furthermore, the optimization method that we describe can be used on other yet uncultivable symbionts, opening new technical opportunities in the field of host-microbes interactions.

## INTRODUCTION

Insects frequently maintain symbiotic relationships with vertically transmitted bacterial partners that live within their body, called endosymbionts. Some endosymbionts provide a direct benefit to the host’s development and fertility by complementing its diet. Others grant their host with a conditional benefit that arises only in given contexts, for example by providing resistance to heat, parasites, or viruses (1). Deciphering the molecular dialogue that underlies host-endosymbiont interactions is thus of major importance to better understand the physiology and evolution of insects. However, functional studies are often focused on the host side, because nearly all endosymbiotic bacteria are uncultivable, and thus genetically intractable. As a consequence, the bacterial determinants that affect the interaction remain largely unknown. To date, only four endosymbionts, *Sodalis glossidinus* (tsetse flies), *Arsenophonus arthropodicus* (louse flies), *Serratia symbiotica* (aphids), and *Hamiltonella defensa* (aphids), have been cultivated in cell-free media (2–5). Systems of coculture with insect cell lines have also been used successfully for some endosymbionts (6), but such techniques are difficult, and they do not always allow genetic engineering.

The *Spiroplasma* genus comprises diverse bacteria, including commensal, pathogenic, and mutualistic species, most of them being obligate associates with arthropod or plant partners (7). *Spiroplasma* cells are long, helical, and devoid of a cell wall. Extensively studied species include pathogens of crustaceans (8), insects (e.g., the bee pathogen *Spiroplasma melliferum* [9]), and plants. Plant pathogens proliferate in phloem and are vectored by phloem-feeding insects (10–12). Some, notably *Spiroplasma citri*, can be grown *in vitro* and are amenable to genetic studies. In addition to strains that are infectious and transmitted horizontally between hosts, many *Spiroplasma* are facultative inherited endosymbionts of insects (i.e., with transovarial transmission).

Along with *Wolbachia*, *Spiroplasma* bacteria are the only known inherited symbionts of *Drosophila* (13). By far the best-studied species (and strain) is *Spiroplasma poulsonii* MSRO (MSRO for melanogaster sex ratio organism), which infects *Drosophila melanogaster* and is the focus of this study. As other facultative endosymbionts, *S. poulsonii* is transmitted vertically with high efficiency, causes reproductive manipulation (male killing), and confers protection to its *Drosophila* host against parasitoid wasps (14, 15).

Taking advantage of the genetic tools available in *Drosophila*, current work has started to investigate the molecular mechanisms underlying *Drosophila*-*Spiroplasma* symbiosis (16–19). The study of the bacterial determinants, however, has been hampered by the fact that the endosymbiont was unculturable. To expand the toolbox with which to study this endosymbiosis, we designed a method to optimize the Barbour-Stoenner-Kelly H (BSK-H) medium (21) so it allows a sustainable *in vitro* culture of *Spiroplasma*. We also resequenced the *S. poulsonii* MSRO genome in order to provide a complete draft of the chromosome, as well as the first complete sequence of a natural plasmid in this species. By comparing the transcriptome of the bacterium *in vitro* and in the host, we identified genes potentially involved in the interaction with the host.

## RESULTS

### Design and optimization of a culture medium for *S. poulsonii.*

**FIG 1:**
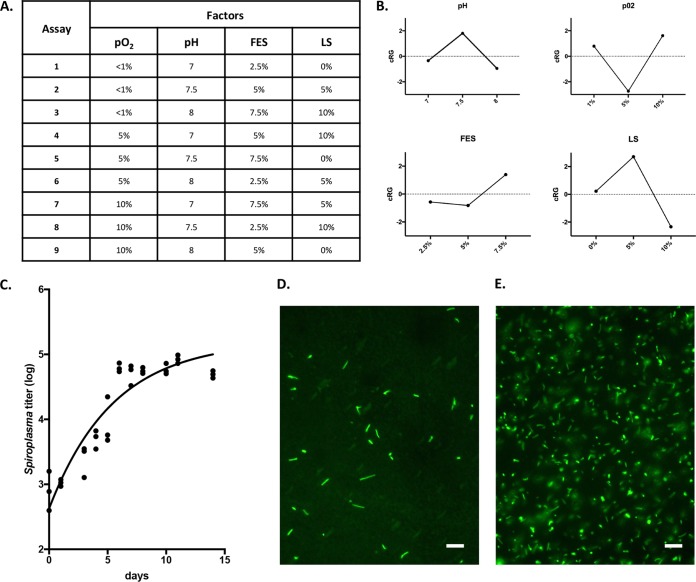
(A) Orthogonal matrix of assays for optimization of the BSK-H-spiro medium. Each assay was independently repeated three times. pO_2_, partial pressure in O_2_; FES, fly extract supplementation; LS, lipid supplementation. (B) cRG values computed from the assays for each factor. (C) Growth curve of *S. poulsonii* in BSK-H-spiro medium at 25°C under 10% O_2_ and 5% CO_2_. Each point represents one quantitative PCR (qPCR) measurement of *Spiroplasma* titer in one repetition. The line represents a one-phase exponential fit computed on three independent repetitions. (D and E) Freshly diluted (D) and 2-week-old (E) cultures stained with Syto9. Bars, 10 µm.

Unlike pathogenic *Spiroplasma* species, *S. poulsonii* has a partially degenerated genome (20), leading to poor adaptability to environmental changes. *In vitro*, it results in the inability of *S. poulsonii* to grow in culture media designed for pathogenic *Spiroplasma*, such as SP4 medium (60). We thus developed a new medium using as a starting point the commercial medium Barbour-Stoenner-Kelly H (BSK-H). This standardized complex medium has been designed for the culture of the spirochete *Borrelia burgdorferi* (21) and is enriched in nutrients that are predicted to be required by *S. poulsonii* based on its genome. The base medium allows for survival of *S. poulsonii* for several days but does not sustain its growth. To optimize the medium composition, we elaborated an experimental design to assess the effects of four factors on growth: pH, partial pressure in O_2_ (pO_2_), fly extract supplementation (FES), and lipid supplementation (LS). Three levels for each factor were cross-tested against each other following an orthogonal array of assays in BSK-H medium (Fig. 1A). To penalize factor levels that bring in high variability, we computed a growth indicator called contribution to reproducible growth (cRG; mathematical details in Materials and Methods). The analyses of cRG values predicted the best medium to be BSK-H medium supplemented with 7.5% fly extract and 5% lipid mix at pH 7.5 and under 10% pO_2_ (Fig. 1B). Experimental validation of this predicted best medium, named BSK-H-spiro, yielded sustained growth of *S. poulsonii* for 6 to 7 days before the bacterial titer reaches a plateau (Fig. 1C). The growth was confirmed by microscopy observations (Fig. 1D and E). *S. poulsonii* culture in BSK-H-spiro medium could be maintained for more than a year by twofold or threefold dilutions in fresh medium every week. Repeated greater dilutions progressively lead to the collapse of the culture (see Fig. S1 in the supplemental material). Interestingly, the culture can also be frozen at −80°C for more than a year and revived without adding any cryoprotectant. This singularity is likely due to their lack of a cell wall that makes them more deformable, thus more resilient to freezing-induced mechanical stress.

10.1128/mBio.00024-18.2 FIG S1  *S. poulsonii* titer in BSK-H-spiro medium diluted weekly with fresh medium. Each bar represents the average titer in three independent replicate experiments. Error bars represent standard deviations. *S. poulsonii* was not detected by qPCR from passage 5 on when the culture was diluted by 10. Download FIG S1, TIF file, 0.4 MB. Copyright © 2018 Masson et al. 2018 Masson et al. This content is distributed under the terms of the Creative Commons Attribution 4.0 International license.

The BSK-H-spiro medium allowed a doubling time of around 30 h with no difference between a 1-month-old culture and a 1-year-old culture. Infection of naive flies with the culture resulted in a 100% transmission success (24/24 flies) for the 1-month-old culture and 96% infection success (23/24 flies) for the 1-year-old culture, although the amount of bacteria injected (10^2^/fly) is lower than the amount usually injected during hemolymph transfer infections (10^4^/fly). All culture-infected flies transmitted the bacteria to their offspring and displayed a male-killing phenotype, suggesting that prolonged *in vitro* culture does not significantly alter the host-interacting abilities of *S. poulsonii*.

### *S. poulsonii* genome sequence update.

**FIG 2:**
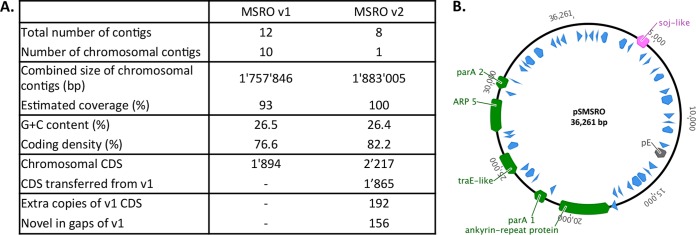
(A) Comparison between the first draft genome (version 1 [v1]) of *S. poulsonii* MSRO (20) and this work (version 2 [v2]). CDS, coding sequence. (B) Graphic map of contig 7 after circularization (plasmid pSMSRO). Blue unnamed arrows are hypothetical protein-coding sequences without annotation. Green arrows are annotated genes. The pink arrow indicates a pseudogene, and the gray arrow indicates the coding sequence of the *Spiroplasma* plasmid replication protein pE.

The *S. poulsonii* MSRO genome was first sequenced and annotated in 2015 (20). However, the presence of repeated sequences complicated the assembly of this draft genome that covered only 93% of the estimated chromosome size. Furthermore, there was doubt about the nature of two extrachromosomal contigs that could have been either plasmids or misassembly products. To complete the genome sequence, we took advantage of recent upgrades in PacBio technology and performed a second sequencing. The new assembly produced eight contigs, including a large contig of 1.8 Mb corresponding to the full circular chromosome of *S. poulsonii*. A total of 2,217 coding sequences (CDS) were identified, of which 1,865 are identical to those in the first assembly prediction (Fig. 2A). Seven smaller contigs were also produced, of which one (contig 7) could be circularized. We aligned this contig to the reference sequences of plasmids from *S. citri* and *Spiroplasma kunkelii*, for which plasmids have been well characterized (Fig. S2). The alignment revealed two conserved synteny blocks between contig 7 and the references. We also detected a coding sequence with 87% homology to the plasmid replication protein sequence pE, proven to code for a plasmid replication protein on *S. citri* plasmids (22). The presence of those genes on the circular sequence of contig 7 strongly suggests that it is the first full sequence of a plasmid in group IV of *Spiroplasma*, to which *S. poulsonii* belongs, and hereafter called pSMSRO (Fig. 2B). The analysis of the remaining extrachromosomal contigs did not allow circularizing any of the contigs or detecting any conserved genes with other *Spiroplasma* plasmids, although we cannot exclude the possibility that other plasmids were present but not detected in the sequencing data.

10.1128/mBio.00024-18.3 FIG S2  Multiple alignment of contig 7 with reference plasmid sequences of *S. citri* and *S. kunkelii* using the MAUVE progressive algorithm. Conserved synteny blocks (red arrows) contain an *ARP* operon (green block) and a *traE* operon (blue block). Download FIG S2, PDF file, 0.7 MB. Copyright © 2018 Masson et al. 2018 Masson et al. This content is distributed under the terms of the Creative Commons Attribution 4.0 International license.

This new draft confirmed the metabolic landscape already described by Paredes et al. (20), with no obvious difference regarding the presence or absence of metabolic genes. However, the full coverage and extended annotation of the new draft allowed us to compile a comprehensive list of *Spiroplasma poulsonii* virulence factors (Table 1). Five virulence factors were initially reported in the first genome: two spiralins (Spiralin A and B), a chitinase (ChiD), a cardiolipin synthase (Cls), and a glycerol-3-phosphate oxidase (GlpO). Spiralin A is found in other *Spiroplasma* species, including *S. citri*, while Spiralin B is found only in *S. poulsonii* (20). We found a third gene coding for a spiralin-like protein, Spiralin C, which shares only 15% homology with *spiA* and *spiB* but has a conserved spiralin domain. We also identified a group of five genes coding for adhesion-related proteins (ARPs) that were present in the first draft but misannotated. Intriguingly, these genes include one sequence located on pSMSRO, but also four sequences located on the chromosome, while all *S. citri* ARPs are extrachromosomal (23). A sixth chromosomal ARP pseudogenized by an insertion sequence was identified, as well as two shorter genes partially homologous to ARPs. We also identified a gene containing a *Clostridium* epsilon toxin (Etx) conserved domain. Etx are major toxins of *Clostridium perfringens* and cause a variety of symptoms in mammals, including brain damage (24). The results of our genome analysis confirm the presence of five sequences coding for ribosome-inactivating proteins (RIPs), that were initially identified by Hamilton et al. (25). Last, the plasmid bears a coding sequence for an ankyrin repeat protein (Ank). Ank repeats are found in many virulence effector proteins (26). Remarkably, they are widely found in the genome of *Wolbachia*, another widespread endosymbiont that manipulates insect reproduction, and their large number and diversity suggest that they could play a crucial role in host-symbiont interactions (27).

**TABLE 1:** Virulence factors of *S. poulsonii*

Family and virulence factor	GenBank locus tag	Contig	Coordinates	Signal peptide	TM domain^a^	Predicted location	Reference comment^b^
Spiralins							
SpiA	SMSRO_SF013140	1	1005468–1004767	Yes	No	Membrane	A
SpiB	SMSRO_SF009660	1	753920–754717	Yes	No	Membrane	A
SpiC	SMSRO_SF015890	1	1203572–1204045	No	No	Unknown	C
Adhesion-related proteins^c^							
SpARP1	SMSRO_SF002520	1	205842–206939	Yes	1	Membrane	C
SpARP2	SMSRO_SF011850	1	908030–909277	Yes	Unsure	Membrane	C
SpARP3	SMSRO_SF022680	1	1731722–1730625	Yes	1	Membrane	C
SpARP4	SMSRO_SF024450	1	1870575–1871513	Yes	1	Membrane	C
SpARP5	SMSRO_SFP00390	7	12713–12147	Yes	1	Membrane	D
Metabolic genes							
Cls	SMSRO_SF001010	1	81414–82952	No	3	Membrane	A
ChiD1	SMSRO_SF008450	1	671704–672774	Yes	No	Secreted	A
ChiD2	SMSRO_SF013110^d^	1	1002344–1002357				C
GlpO	SMSRO_SF018440	1	1400479–1401657	No	No	Cytosol	A
Toxins							
RIP1	SMSRO_SF016530	1	1253115–1254512	Yes	No	Secreted	B
RIP2	SMSRO_SF018820	1	1438476–1439966	Yes	No	Secreted	B
RIP3	SMSRO_SF023880	1	1820456–1821802	Yes	No	Secreted	B
RIP4	SMSRO_SF020720	1	1584448–1585794	Yes	No	Secreted	B
RIP5	SMSRO_SF003660	1	293319–294665	Yes	No	Secreted	B
ETX-like	SMSRO_SF021610^e^	1					C
Ankyrin repeat	SMSRO_SFP00290	7	6975–9461	Yes	No	Secreted	D

^a^ The presence or absence of a transmembrane domain and if present, the number of transmembrane domains.

^b^ Reference comments give additional information about genes as follows: A, the gene was detected and annotated by Paredes et al. (20); B, the gene was detected by Paredes et al. (20) and annotated and discussed by Hamilton et al. (25); C, the gene was detected by Paredes et al. (20) but was not annotated and/or not discussed; D, the gene was detected and annotated in this work for the first time.

^c^ SpARP1, *S*. *poulsonii* ARP1.

^d^ This gene was pseudogenized.

^e^ The gene structure of this gene was unclear.

### Transcriptome analysis of *S. poulsonii* in culture versus in host.

To detect genes involved in *S. poulsonii* interaction with its host, we compared the transcriptome of *S. poulsonii* collected from *Drosophila* hemolymph to the transcriptome of *S. poulsonii* cultured *in vitro* for 2 months. This reference transcriptome produced by pooling transcripts detected in both conditions contains 1,491 transcripts. Of the reads, 97.18% mapped to the chromosome and 1.74% mapped to contig 7, while no significant signal was detected for any other extrachromosomal contig. This supports the hypothesis that contig 7 is a plasmid of *S. poulsonii*, while other extrachromosomal contigs are misassembly products. The most expressed gene under all conditions is *spiB*, followed by housekeeping genes related to cell division, transcription, and translation.

**FIG 3:**
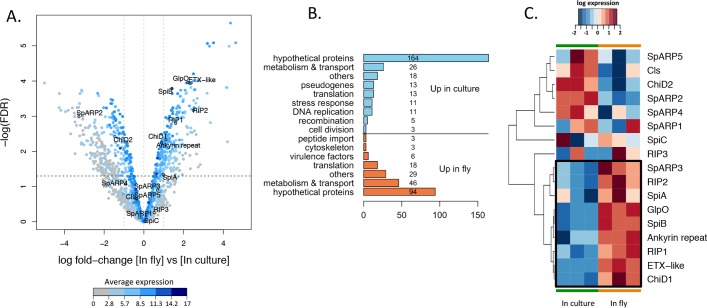
(A) Volcano plot of differential gene expression of *S. poulsonii* in host versus in culture. Each point represents the average value of one transcript in three replicate experiments. The expression difference is considered significant for a log_2_ fold change of ≥1 (outer light gray broken vertical lines) and for a *P* value of ≤0.05 [−log(FDR) of ≥1.3, dark broken horizontal line]. Points are colored according to their average expression in all data sets. Names and outlined points represent virulence factors. FDR, false-discovery rate. (B) Manual clustering of the transcripts differentially expressed by *S. poulsonii* in the fly versus in the culture. The numbers of sequences in the different categories are indicated on the bars or to the right of the bars. (C) Heatmap of *S. poulsonii* virulence gene expression. Each column represents the value for one replicate experiment in culture or in the fly. The colors represent the log_10_ level of expression in the corresponding experiment. The cluster of genes that are induced when *S. poulsonii* is in the host (versus *in vitro*) is shown enclosed in a black box. SpARP5, *S. poulsonii* ARP5.

Pairwise comparison between the two experimental conditions identified 465 genes differentially expressed, 201 of the genes being more transcribed in the host, while 264 were significantly more expressed in culture (Fig. 3A). A total of 258 (55%) of the differentially expressed genes were annotated only as “hypothetical proteins” and were not further accounted for in the analysis. Genes that were identified by homology but whose function was very general or unclear were grouped in the category “others.” The remaining sequences have been manually clustered according to their predicted function (Fig. 3B). A majority of identified genes that were found differentially expressed were associated with metabolic pathways and metabolite transport, probably as a consequence of differences between the composition of the medium and the fly hemolymph. Aside from this, a large cluster of genes was related to ribosome assembly and translation, including ribosome structural proteins, tRNA ligases, and translation regulators. Some members of this cluster were found upregulated in culture, while others were upregulated in the fly, suggesting that the switch of environment triggers a qualitative change in the translational activity of the bacterium. The *in vitro* culture data set also showed enrichment in transcripts related to DNA replication and cell division, consistent with a doubling time of around 30 h *in vitro* versus 170 h in the adult fly (28). Interestingly, transcripts involved in DNA recombination were also enriched in *S. poulsonii* grown in culture, including *ruvA*, *ruvB*, *recR*, and *recU*, as well as genes belonging to the *comEC* family. The *comE* operon contributes to the natural competence in *Bacillus subtilis*, ensuring the binding and uptake of transforming DNA (29), which suggests that *S. poulsonii* might be naturally competent.

Last, several differentially expressed sequences were identified as pseudogenes resulting either from a frameshift mutation or from the insertion of a mobile element in the coding sequence that causes the protein to be truncated. The active transcriptional regulation of these genes suggests a recent pseudogenization, possibly as a consequence of *S. poulsonii* switching from a free-living lifestyle to an endosymbiotic lifestyle.

Virulence factors could be classified in two clusters depending on their expression profile (Fig. 3C). *spiA*, *spiB*, *RIP1*, *RIP2*, *ank*, *etx*, *glpO*, and *chiD1* have lower expression levels in culture than in the host, pointing to their role in host-symbiont interaction. Other virulence genes do not display a significant change in their expression level and have low average levels of expression. Such genes include *spiC*, *RIP3*, *SpARP1*, *SpARP4*, *SpARP5*, *chiD2*, and *cls*. *SpARP2* is the only virulence gene that is expressed at higher levels in culture than in the fly, and *RIP4* and *RIP5* as well as *SpARP3* are not detected at all in the transcriptome, implying that they might be pseudogenes, resulting from a duplication of the coding sequence without the regulatory upstream sequence.

Finally, a gene encoding a ferritin, a protein involved in iron sequestration, was expressed at a higher level in *S. poulsonii* extracted from *Drosophila* than in *S. poulsonii* grown in culture, suggesting that iron availability could be a proliferation-limiting factor along with lipids and glucose availability (17, 20).

## DISCUSSION

We developed the first reliable method to culture endosymbiotic *S. poulsonii* from *Drosophila* in a cell-free medium. This is an important step forward because cultivation is a prerequisite for addressing functional questions on the regulation of this symbiosis via genetic manipulation of the bacterial partner. While pathogenic *Spiroplasma* bacteria, such as *S. citri*, can be easily cultivated in standard growth media, no suitable medium was available to grow any endosymbiotic *Spiroplasma* outside their hosts. This was not for a lack of trying: much work was done in the 1980s to attempt to set up a culture medium for *S. poulsonii*, until one paper reported in 1986 the successful cultivation of a *Drosophila Spiroplasma* in a cell-free medium (30). According to the authors, the critical factor was to supply *Spiroplasma* with growing insect cells in the course of primary isolation, followed by a succession of passages that allowed for *Spiroplasma* to adapt to an insect cell-free medium. Unfortunately, this work could not be repeated despite several attempts from various laboratories. *Spiroplasma* thus remained uncultivable in practice.

An important difference between previous attempts and the present work is that crucial requirements of the symbiont were addressed based on very recent discoveries about *S. poulsonii* physiology. The need of *S. poulsonii* for host lipids to synthesize its membrane, for example, was demonstrated only a few years ago (17, 20), and lipid supplementation turned out to be crucial to promote growth *in vitro*. Another difference was supplementing the medium with fly extract, which was also not undertaken in previous works. Since the unsupplemented medium already contains glucose, essential amino acids, lipids, and vitamins needed by the bacteria, we assume that the growth improvement observed with fly extract supplementation might come either from a fly hormone or neurotransmitter or from a nonorganic growth factor available in the fly. A possible candidate would be iron, as suggested by the overexpression of a ferritin-like coding gene by *S. poulsonii in vitro*, or another metallic ion, not present in sufficient quantities in the BSK-H base medium (31). Importantly, starting with a high density of bacteria seems to be a key point for the successful establishment of the culture. Beginning with an amount of infected hemolymph that is too small does not allow the culture to thrive, and repeated strong dilutions lead to a collapse of the culture. This suggests the existence of a density threshold below which *S. poulsonii* growth is inhibited. The exact mechanism leading to this inhibition remains elusive however, as the genome analysis did not highlight any quorum-sensing system.

This work also allowed an initial comparison between the *in vivo* transcriptome and the *in vitro* transcriptome of *S. poulsonii*, identifying genes that are overexpressed when the bacterium is in contact with its host. Membrane proteins are particularly interesting, as they are associated with host infection in pathogenic *Spiroplasma*. *S. citri* spiralin for example acts as a lectin and binds to insect host’s glycoproteins to invade cells (32, 33). Its expression is downregulated when the bacterium is in its plant hosts compared to *in vitro* culture, while its expression is not altered in the insect and in culture (34). In *S. poulsonii*, *spiA*, the closest homologue to the *S. citri spiralin* gene, is only slightly upregulated in the insects, while *spiB*, which is found only in *S. poulsonii*, is strongly upregulated. This points to a function of SpiB that is specific to endosymbiosis, possibly related to the bacterial entry in the oocyte during vertical transmission. ARPs are also major lipoproteins involved in *S. citri* transmission (35, 36), turning out to be essential for insect cell invasion but nonessential for transmission from insects to plants (37). In *S. poulsonii*, few predicted ARPs have a complete and functional sequence, and their expression is not differentially regulated *in vitro* compared to in the host. *S. citri*, as a strictly horizontally transmitted pathogen, could require diverse ARPs to infect new hosts efficiently. *S. poulsonii* on the other hand is mostly vertically transmitted, although horizontal transmission to new host is possible notably via ectoparasite vectors (38, 39). ARPs in this species could thus be less diverse because of its more limited host range. The chromosomal location of most *S. poulsonii* ARPs (rather than extrachromosomal as in *S. citri* [23]) also reflects a lower ability of these genes to be horizontally transferred. This could reflect the fixation of this gene family during the coevolution of vertically transmitted *Spiroplasma* with its host.

Several genes coding for toxins are overexpressed in the host compared to *in vitro*, including ribosome-inactivating proteins (RIPs) and two yet uncharacterized toxins (Ank and Etx). RIPs are involved in the protection of *Drosophila* against nematodes and parasitoid wasps by selectively inactivating the 28S rRNA of the parasites (25, 40). The upregulation of *RIP1* and *RIP2* when *S. poulsonii* is in the host compared to *in vitro* suggests that RIPs could have a function in host-symbiont interactions, regardless of parasite infections, possibly in male killing. Etx might be involved in the neuronal symptoms, notably tremors and dopaminergic neuron degeneration observed in *Spiroplasma*-infected flies when the flies are old (28). We also cannot exclude the possibility that it functions as a defensive agent against parasites, which would explain the transcription increase when *S. poulsonii* is in the insect compared to *in vitro*, although the toxicity of Etx has not yet been investigated in nonmammal models. Further functional studies will be necessary to assess the exact function of these toxins in the *S. poulsonii-Drosophila* interaction.

It is noteworthy that 2 out of 18 identified virulence genes (*SpARP5* and *ank*) are located on a plasmid, which indicates that extrachromosomal DNA may play an important role in *S. poulsonii-Drosophila* interactions. Variability in plasmid presence and/or copy number in *S. poulsonii* strains could be accountable for the variability in the host phenotypes caused by the endosymbiont, including variable male-killing penetrance.

In conclusion, the method described in this work is the first protocol to allow cultivation of an endosymbiotic *Spiroplasma* in a cell-free medium in almost 30 years. The technical approach that was used to design the BSK-H-spiro medium can be adapted to optimize a medium for other uncultivable bacteria, for which a favorable physicochemical environment can be partially predicted. The expression of *comE* indicates that *S. poulsonii* might be naturally competent and the transcriptional regulation findings (up- or downregulation) observed with recombination-related genes suggest that knockout mutants by homologous recombination might be possible despite *recA* pseudogenization (20), as in pathogenic *Spiroplasma* species (41, 42). Eventually, several bacterial genes were predicted to have a key function in the interaction between *S. poulsonii* and its host, including virulence factors. These genes are thus priority candidates for further investigation upon the development of genetic tools to modify *S. poulsonii* in order to unravel their precise function. Coupled with the powerful genetic tools available on the *Drosophila* side, the development of genetic tools to modify *S. poulsonii* will be a major achievement in the field of symbiosis, as it will provide the first insect model where both the host and the endosymbiont are readily transformable.

## MATERIALS AND METHODS

### *Spiroplasma* stock.

We used a wild-type Oregon-R (OR^R^) fly stock that has been cured of *Wolbachia* by antibiotic treatment and infected by the *Spiroplasma poulsonii* MSRO strain Uganda (28, 43). The stock has been maintained in the lab for several years between these treatments and the experiments.

### Cell-free culture medium design.

The medium basis was the Barbour-Stoenner-Kelly H medium (BSK-H) without l-glutamine from BioSell (Feucht bei Nürnberg, Germany). BSK-H medium from Sigma has also been used successfully. The design of an orthogonal array of growth assays was based on the choice of four factors (pH, partial pressure in oxygen [pO_2_], fly extract supplementation [FES], and lipid supplementation [LS]) that were *a priori* expected to affect *Spiroplasma* growth significantly. The use of an orthogonal array allows the extraction of relevant information from a reduced number of factor level combinations rather than from all possible combinations. For each factor, three levels were arbitrarily chosen around an expected optimal value (e.g., pH 7.5 as the expected optimal value, pH 7, 7.5, and 8 as the tested levels). Cultures were started from hemolymph extracted from the thorax of 1-week-old infected flies by aspiration with a Nanoject II nanoinjector (Drummond Scientific). A preculture was launched 1 week prior to the experiment by adding 8 µl of hemolymph to 3.2 ml of BSK-H medium plus 5% fly extract (1,000 to 5,000 bacteria/µl) without agitation. Aliquots of 100 µl of preculture were then frozen at −80°C before use. For each assay, aliquots were centrifuged for 40 min at 2,000 relative centrifugal force (rcf) at 18°C, and pellets were resuspended in 200 µl of medium. Aliquots (10-µl aliquots) were taken 1 day and 7 days later for growth assessment by quantitative PCR. A linear regression on the log-transformed measures between day 1 and day 7 was computed for each level of each tested factor, and the slope of the regression was used as a growth rate measurement. Three independent replicates were made for each medium testing. Since some combinations of factors yielded high variability in growth, we analyzed the data with a statistical approach inspired from the Taguchi method (44, 45). To penalize factor levels that entail a high variability in growth, a “reproducible growth” (RG) parameter was computed, $\text{RG}=10\times \log\left({\bar{S}}^{2}\right)-10\times \log\;[1+3\times \left(\sigma /\bar{S}{)}^{2}\right]$ where *S̄* is the average slope with the considered level of the considered factor and σ is the standard deviation. The contribution of one level to the reproducible growth (cRG) was calculated as cRG = RG_considered level _− RG_all levels_. For each factor, the level with the highest cRG was selected as the optimal value. An experimental validation was then performed in a medium bringing together the best levels for each of the four factors, hereafter designed as BSK-H-spiro, following the same protocol as for the optimization assays. Three independent replicates were made for the validation assay. Protocols for preparing the fly extract, lipid mix, and BSK-H-spiro medium are detailed in Text S1 in the supplemental material. The media of freshly started cultures were completely renewed every three or four passages until at least passage 12, by centrifugation for 20 min at 12,000 rcf at 18°C and replacement of the used medium (supernatant) by an equivalent amount of fresh medium. These replacements are a necessary adaptation step that becomes unnecessary for older cultures. Long-lasting cultures were maintained by a weekly threefold dilution in fresh culture medium. All experiments and cultures were performed at 25°C, which is the temperature at which *Drosophila* infected stocks are routinely maintained, and yet close to the 26°C predicted optimal temperature for *S. poulsonii* (46).

10.1128/mBio.00024-18.1 TEXT S1  Recipes for fly extract, lipid mix, and BSK-H-spiro medium. Download TEXT S1, PDF file, 0.05 MB. Copyright © 2018 Masson et al. 2018 Masson et al. This content is distributed under the terms of the Creative Commons Attribution 4.0 International license.

### Culture density measurement.

For DNA extraction for quantitative PCR, bacteria were lysed by osmotic shock by adding 400 µl of distilled water to 10 µl of culture and heated at 95°C for 15 min. This simple method ensures an efficient yield from a small amount of initial bacterial material. DNA was then used for quantitative PCR as described before (47) with primers amplifying a 300-bp fragment of the 16S rRNA gene (primer forward, 5′-TACATGCAAGTCGAACGGGG-3′; primer reverse, 5′-CTACTGCTGCCTCCCGTAG-3′). Microscopic observation was performed as previously described (28).

### Fly infections.

One-week-old female Oregon flies were infected from the *S. poulsonii* culture by an injection of 23 nl of a dense culture (1 week after the latest dilution with fresh medium) with a Nanoject II nanoinjector (Drummond Scientific). Flies were allowed to recover from the injection in a tube with fresh medium in the absence of males for 1 day. Each female fly was then coupled with a male, and each couple was isolated in a tube in order to monitor the infection status of the progeny of single flies. Couples were flipped onto fresh medium every 2 or 3 days. The eggs laid for the first week following mating were discarded. The progeny was screened for *S. poulsonii* infection 1 week after hatching by Syto9 staining as previously described (28).

### Genome sequencing and analysis.

*S. poulsonii* DNA was extracted from fly hemolymph as previously described (20). Processing of the samples was performed in the University of Lausanne Genomic Technologies Facility. The DNA was sheared in a Covaris g-TUBE (Covaris, Woburn, MA, USA) to obtain 20-kb fragments. After the DNA was sheared, the size distribution of the DNA fragments was checked on a Fragment Analyzer (Advanced Analytical Technologies, Ames, IA, USA). Five micrograms of the sheared DNA was used to prepare an SMRTbell library with the PacBio SMRTbell template prep kit 1 (Pacific Biosciences, Menlo Park, CA, USA) according to the manufacturer’s recommendations. The resulting library was size selected on a BluePippin system (Sage Science, Inc., Beverly, MA, USA) for molecules larger than 20 kb. The recovered library was sequenced on one SMRT cell with P6/C4 chemistry and MagBeads on a PacBio RSII system (Pacific Biosciences, Menlo Park, CA, USA) in a 240-min movie. Assembly was performed with HGAP (hierarchical genome assembly process) version 2 from the PacBio smrtpipe (v2.3.0). Circularization of main contig 1 was performed using Amos (v3.1.0; Amos Consortium; http://amos.sourceforge.net). Plasmid contig 7 was refined using the PacBio read data from Paredes et al. (20) with Quiver version 1. Genome annotation was performed with Prokka v 1.11 (48) using parameters --addgenes --genus Spiroplasma --species poulsonii --gcode 4 --rawproduct –rfam --rnammer). The Nucmer tool (Mummer suite v3.23 [49]) was used to align coding sequences (CDS) from the annotated version (accession no. JTLV01000000) to the new annotation. Some gene product annotations were refined using NCBI PSI-blast tool. Multiple alignments of *Spiroplasma* plasmids have been performed using MAUVE version 2.4.0 (50).

### RNA sequencing and analysis.

RNA was extracted from (i) the hemolymph of 30 1-week-old infected flies and (ii) 20 ml of 4-month-old *in vitro* culture pelleted by 30 min of centrifugation at 16,000 ×  *g* by the TRIzol method following the manufacturer’s instructions. Three independent replicates were prepared for each condition. Libraries were prepared using the Illumina Truseq RNA kit and sequenced on an Illumina HiSeq 2000 system at the University of Lausanne Genomic Technologies Facility. Purity-filtered reads were adapters and quality trimmed with Cutadapt v.1.8 (51). Reads matching to rRNA sequences were removed with fastq_screen v. 0.9.3 (Babraham Bioinformatics; http://www.bioinformatics.babraham.ac.uk/projects/fastq_screen/). Remaining reads were further filtered for low complexity with reaper v. 15-065 (52). Reads were aligned against the *Spiroplasma poulsonii* MSRO (v2) genome using STAR v. 2.5.2b (53). The number of read counts per gene locus was summarized with htseq-count v. 0.6.1 (54) using *Spiroplasma poulsonii* MSRO (v2) gene annotation. The quality of the transcriptome sequencing (RNA-seq) data alignment was assessed using RSeQC v. 2.3.7 (55). Statistical analysis was performed for genes in R version 3.4.1 (56). Genes with low counts were filtered out according to the rule of one count per million (cpm) in at least one sample. rRNA and tRNA gene counts were discarded. Library sizes were scaled using TMM normalization with EdgeR package version 3.18.1 (57) and log cpm transformed with limma voom function, limma package version 3.32.5 (58). Differential expression was computed with limma (59) by fitting the samples into a linear model and performing “in fly” versus “in culture” comparison. Moderated *t* test was used, and adjusted *P* values were computed by the Benjamini-Hochberg method, controlling for the false-discovery rate.

### Accession number(s).

The genome with DDBJ/EMBL/GenBank accession no. JTLV00000000 was updated. The genome version described in this paper has accession no. JTLV02000000. The RNA-seq differential expression analysis can be found in Data Set S1.

10.1128/mBio.00024-18.4 DATA SET S1  Detailed differential expression analysis of *S. poulsonii* transcriptome in the host versus *in vitro*. Download DATA SET S1, TXT file, 0.4 MB. Copyright © 2018 Masson et al. 2018 Masson et al. This content is distributed under the terms of the Creative Commons Attribution 4.0 International license.
