# Supplementary figures and images for: In Vitro Culture of the Insect Endosymbiont Spiroplasma poulsonii Highlights Bacterial Genes Involved in Host-Symbiont Interaction
Source: mBio. 2018 Mar 20;9(2):e00024-18. doi: 10.1128/mBio.00024-18 (PMC5874924; doi:10.1128/mBio.00024-18)

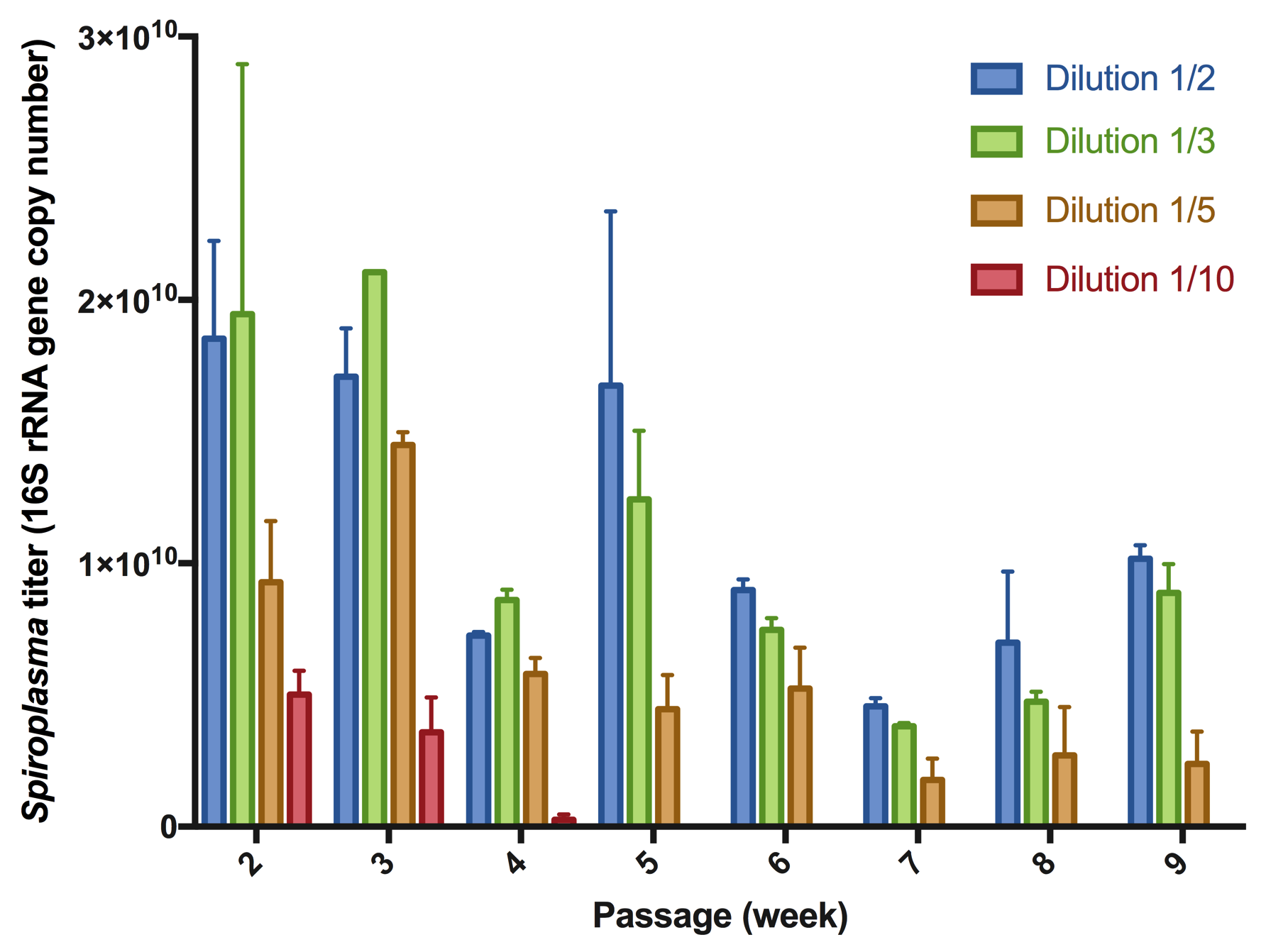

Supplement: FIG S1 [file mbo002183782sf1.tif]

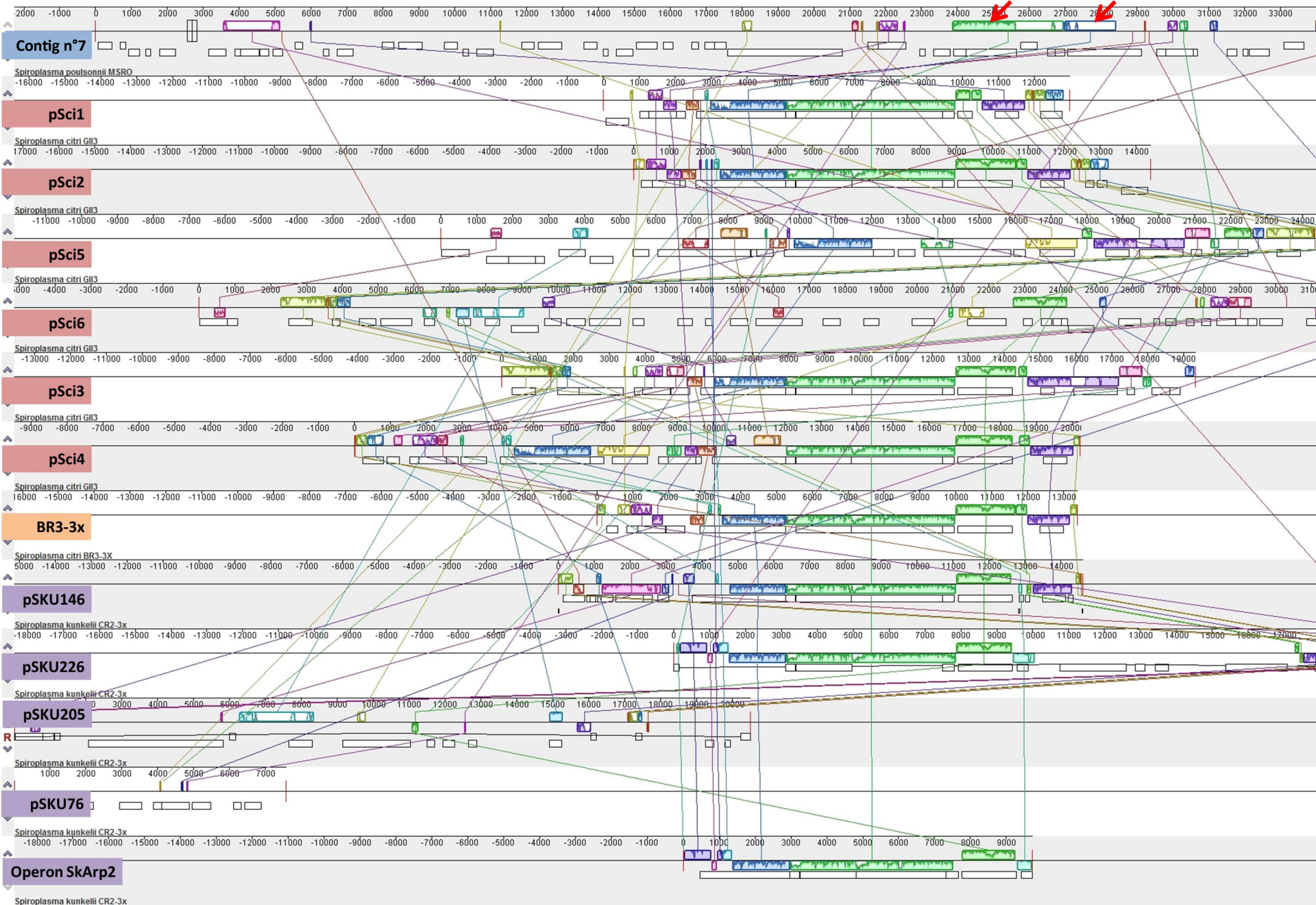

Supplement: FIG S2 [file mbo002183782sf2.pdf]
