# Supplementary material for: In Vitro Culture of the Insect Endosymbiont Spiroplasma poulsonii Highlights Bacterial Genes Involved in Host-Symbiont Interaction
Source: mBio. 2018 Mar 20;9(2):e00024-18. doi: 10.1128/mBio.00024-18 (PMC5874924; doi:10.1128/mBio.00024-18)
Supplement: TEXT S1 [file mbo002183782s1.pdf]

Fly extract preparation

- Collect 1 to 7-days-old flies infected with *Spiroplasma.* Flies can be stored at -20°C for later use
- Add 30 mL of BSK-H medium without L-glutamin (BioSell) for 6 g of flies and crush thoroughly with a Dounce tissue grinder
- Incubate 20 minutes at 56°C
- Centrifuge 15 minutes at 3000 g and collect the supernatant
- Filter at 0.45 µm
- Filter at 0.22 µm
- Store at -20°C

Lipids mix preparation

- Dissolve 10 mg of cholesterol, 5 mg of palmitic acid and 10 mg of sphingomyelin in 1.8 mL of 100% ethanol preheated at 30°C.
- Add :
    - 100 µL of 1-palmitoyl-2-oleoyl-sn-glycerol (stock at 10 mg/mL in ethanol)
    - 100 µL of 1,2-dioleoyl-sn-glycerol (stock at 20 mg/mL in ethanol)
    - 50 µL of Tween 40
    - 50 µL of Tween 80
    - 5.6 µL of oleic acid
- Vortex thoroughly
- Mix 400 µL of the lipids premix to 19.6 mL of fatty-acid-free BSA 6% to form the lipid mix usable in the BSK-H-spiro medium preparation. Store the premix and the mix at -20°C.

BSK-H-spiro preparation

For 400 mL of medium :

- 314 mL of BSK-H without L-glutamin (BioSell)
- 2 mL of penicillin G 130 mg/mL
- 10 mL of arginin 70 mg/mL
- 24 mL of rabbit serum heat-inactivated
- 30 mL of fly extract (7.5% final)
- 20 mL of lipids mix (5% final)
-

Adjust the pH to 7.5 with HCl and filter at 0.22 µm.

Penicillin G and Arginin stock solutions must be prepared in BSK-H rather than water. Do not autoclave the final medium or any of its components. Store at 4°C.
